# Disengaged: a qualitative study of communication and collaboration between physicians and other professions on general internal medicine wards

**DOI:** 10.1186/1472-6963-13-494

**Published:** 2013-11-25

**Authors:** Merrick Zwarenstein, Kathleen Rice, Lesley Gotlib-Conn, Chris Kenaszchuk, Scott Reeves

**Affiliations:** 1 Centre for Studies in Family Medicine, London, ON, Canada; 2 Department of Health Policy, Management and Evaluation, Faculty of Medicine, University of Toronto, 155 College Street, Suite 425, Toronto M5T 3 M6, Canada; 3 Department of Anthropology, University of Toronto, 19 Russell Street, Toronto M5S 2S2, Canada; 4 Department of Surgery, St. Michael’s Hospital, 30 Bond Street, Toronto, ON M5B 1 W8, Canada; 5 Centre for Addiction and Mental Health, 250 College Street, Toronto, ON M5T 1R8, Canada; 6 Center for Innovation in Interprofessional Education, University of California, San Francisco, CA 94143, USA

**Keywords:** Interprofessional collaboration, Ethnography, General internal medicine

## Abstract

**Background:**

Poor interprofessional communication in hospital is deemed to cause significant patient harm. Although recognition of this issue is growing, protocols are being implemented to solve this problem without empirical research on the interprofessional communication interactions that directly underpin patient care. We report here the first large qualitative study of directly-observed talk amongst professions in general internal medicine wards, describing the content and usual conversation partners, with the aim of understanding the mechanisms by which current patterns of interprofessional communications may impact on patient care.

**Methods:**

Qualitative study with 155 hours of data-collection, including observation and one-on-one shadowing, ethnographic and semi-structured interviews with physicians, nurses, and allied health professionals in the General Internal Medicine (GIM) wards of two urban teaching hospitals in Canada. Data were coded and analysed thematically with a focus on collaborative interactions between health professionals in both i*nterprofessiona*l and *intraprofesional* contexts.

**Results:**

Physicians in GIM wards communicated with other professions mainly in structured rounds. Physicians’ communications were terse, consisting of reports, requests for information, or patient-related orders. Non-physician observations were often overlooked and interprofessional discussion was rare. Intraprofessional interactions among allied health professions, and between nursing, as well as interprofessional interactions between nursing and allied health were frequent and deliberative in character, but very few such discussions involved physicians, whose deliberative interactions were almost entirely with other physicians.

**Conclusion:**

Without interprofessional problem identification and discussion, physician decisions take place in isolation. While this might be suited to protocol-driven care for patients whose conditions were simple and courses predictable, it may fail complex patients in GIM who often need tailored, interprofessional decisions on their care.

Interpersonal communication training to increase interprofessional deliberation may improve efficiency, patient-centredness and outcomes of care in hospitals. Also, electronic communications tools which reduce cognitive burden and facilitate the sharing of clinical observations and orders could help physicians to engage more in non-medical deliberation. Such interventions should take into account real-world power differentials between physicians and other health professions.

## Background

Globally, policymakers have identified interprofessional collaboration and communication as a key means by which to improve the quality and safety of patient care [1-5]. The Institute of Medicine in the United States recommends that contemporary health care teams should be “using all the expertise and knowledge of team members to meet patients’ needs [2] (p. 83).” Health Canada adds that collaborative practice in health care “enhances patient-, family-, and community-centred goals and values, [and] optimizes staff participation in clinical decision making” [6] (p.1). Professional associations agree, with the Royal College of Physicians and Surgeons of Canada listing collaboration as a core competency to be acquired during resident training, [7] and the US National Council of State Boards of Nursing succinctly stating that “collaboration is a professional imperative” [8] (p.15). Increasing support for improving interprofessional collaboration is based upon a growing body of evidence demonstrating that poor communication between health professionals harms patients [9-13]. The changing context of contemporary healthcare imposes further strains on healthcare teams. Aging populations with consumer expectations demand complex and personalised care [14]. Rising health care costs and professional shortages limit hiring and decentralise the delivery of care to an ever wider network of loosely-coordinated professionals, organisations and facilities [15-17]. But even within geographically co-located teams, such as on wards within a teaching hospital, the professionalization of nursing, and the restrictions governing resident training may have isolated team members in professional silos [18,19]. Achieving better outcomes thus requires ever more effort to co-ordinate the growing number of increasingly separated health professions and professionals caring for shared patients [10,18,20-23].

In both the US, and in Canada, efforts to solve these problems in hospital have focused on reducing communication error, by implementing shared electronic medical record (EMR) and computerised physician order-entry (CPOE) systems [17,24,25]. However many problems of coordination and communication may arise from lack of a common cross-team understanding of the care priorities for a specific patient at a specific time and the resulting failure of individual team members to align their activities to those priorities, rather than simple miscommunication. This more subtle failure may cause patient, family professional and team confusion and dissatisfaction, with delays and readmission rather than directly attributable specific adverse events [26-28]. EMRs and CPOE are structured, electronic asynchronous communication tools, unlikely to facilitate common understanding of patient needs and alignment of professional priorities, which may depend on face to face interprofessional deliberation.

We report here the first study in which qualitative fieldwork was undertaken to examine the nature of interprofessional interactions, prior to design of an intervention [29]. Although there is a small body of evidence suggesting promising outcomes from interventions to promote interprofessional deliberations and decision-making in health care, [22-28,30-36] these have been designed intuitively, with no prior investigation of the interprofessional communication problem to be solved. We focus on differences in oral communication patterns among professions, in scheduled and unscheduled contexts, to identify factors which restrict or facilitate deliberative verbal communication, to support the development of interventions to improve interprofessional collaboration in health care.

## Methods

### Design

We employed an ethnographic approach to generate detailed accounts of *intra*professional (meaning within the same medical profession, or between related allied health professions such as occupational therapy and physiotherapy) and *inter*professional (meaning between different medical professions, for instance between medicine and nursing) relationships in health care settings. This qualitative methodology advantageously combines observations and interviews to support a nuanced understanding of social interactions [37] not obtainable through survey methods [38].

### Setting and participants

We studied internal medicine teams in two urban teaching hospitals in Canada. In both hospitals the internal medicine units cared for patients with acute medical issues and often one or more co-morbid conditions. At both sites the units were comprised of four clinical teaching units (CTU), each composed of a rotating team that included a staff physician, medical residents, and medical trainees. The number of medical trainees in each CTU varied at any one time from five to nine but usually included one senior resident, two first year residents and three or four medical students. At site A, the six frontline nurses on a shift were ward-based, as were medical teams. At site B, the 12 frontline nurses per shift along with the medical teams were assigned by patient bed, all sharing a large, multi-room ward.

Each CTU was teamed with a group of allied health professionals. These were permanent or contracted employees of the hospital, some of whom also worked outside the internal medicine wards. In both sites, the allied health professions included a pharmacist, dietician, speech language pathologist, and physical and occupational therapists. It could also include a social worker, nutritionist, patient care manager/case manager, discharge planner, recreational therapist, and chaplain. Allied health professionals also periodically supervised trainees in their respective disciplines.

Daily operations were similar at both sites. Nursing staff met at 7 am for nursing rounds and patient handover and medical trainees met at 8 am for morning case study. Interprofessional rounds began between 9–9:30 am and lasted from 10 to 45 minutes depending on attendance and nature of discussion. For the rest of the day practitioners worked mostly on their profession-specific tasks, engaging in impromptu interprofessional interactions as necessary. Such interactions generally occurred in central spaces, such as the charting room and the nursing station.

### Data collection

Two researchers, an anthropologist and a sociologist, spent one month in each hospital and gathered a total of 155 hours of data. Researchers observed 33 scheduled rounds, and devoted 17 sessions to observing unscheduled ward work and associated interactions. Unscheduled observation sessions took place in common areas of the ward, such as around the nursing station. Observing so many scheduled and unscheduled activities was necessary in order to reach saturation in each of four wards from both hospitals. The observations were exploratory in nature, and initially aimed to understand the patterns and modes of communication and collaboration on the wards, with a focus on those that were interprofessional. The researchers strategically placed themselves in different locations on the ward to observe the various forms of oral, written and electronic communication used by staff. During direct observations, researchers’ interactions with staff were minimal and non-interruptive.

Five hours of work shadowing were undertaken to confirm cross-sectional findings longitudinally. We followed health professionals as they carried out patient care activities, noting interprofessional interactions over the shadowing period. ‘Shadowees’ were selected purposively, to represent the different professions working in the internal medicine wards: two resident physicians, two physiotherapists and a charge nurse. Each shadow session lasted between 30 and 45 minutes.

Interviews were used to enhance and clarify data obtained through observations. Two forms of ethnographic interviewing techniques were used: informal and formal. The former were casual in tone, and used conversation-like questions posed to participants during observation and/or shadow sessions. These questions were generated as they arose during data collection, to probe what the researcher had experienced during their fieldwork. The latter were conducted to gain a deeper understanding of clinicians’ views about interprofessional communication. These interviews were guided by an interview schedule which explored the general nature of communication and collaboration on the wards. However, as data collection and analysis occurred iteratively; questions were continuously refined and expanded to allow the research team to pursue critical areas of inquiry.

In total 47 staff were interviewed, selected through purposive sampling^38^ to represent nursing, allied health professions, medicine, and administration. This included three patient care managers, four case managers, one clinical leader manager, three advance practice nurses, two resource nurses, one charge nurse, one registered nurse, four occupational therapists, six physiotherapists, three social workers, two speech language pathologists, two pharmacists, one dietician, one chief resident physician, two residents, one intern, one staff physician, one nurse professor, and one clerical administrator. These individuals were recruited as required, to achieve the goal of analytical saturation whereby no new themes were emerging from the interviews. The interviews were conducted on the wards, variously in the nursing station, corridors, the charting rooms, and in some private offices of the management staff.

Interviews lasted between 5 and 30 minutes, and were not audio recorded. Researchers wrote scratch notes^38^ during observation and interview sessions, which were anonymized to record only professional roles of the participants. These scratch notes were written-up into detailed field notes by the researchers who collected these data following the data collection sessions. These fieldnotes were loaded into Nvivo qualitative data management software, and were stored on password protected computers. The researchers did not record information about individual patients, did not go into patients’ rooms, and did not have access to patient charts.

### Analysis

Qualitative data were coded thematically^38^. Themes which emerged through this analysis process included interprofessional communications in structured and unstructured contexts, intraprofessional communications in structured and unstructured contexts, communications between nurses and physicians specifically, and communications between physicians and allied health professionals specifically. At least two researchers independently coded and analyzed the same data-set, which they presented to the wider research team for discussion and comparison of analysis.

### Ethics

Ethical approval was received from the University of Toronto Health Sciences Research Ethics Board, as well as the ethics boards of both hospitals, on condition of deidentification of the data collected. We obtained verbal consent from participants prior to observing or interviewing, and for close shadowing, written consent.

## Results

Interprofessional interactions:

In 155 hours of data-collection we observed few interprofessional communications in which two or more health professions discussed options in order to reach a shared decision for a patient. Indeed, we found only one example from our data set that shows both a physician and members of several other health professions engaging in deliberative interprofessional communication regarding a patient (Table 1, data extract 1).

**Table 1:** Scheduled interprofessional interactions

1	[The doctor reports on the patient]. The patient’s main issue is urological, and has had a consult, and needs home care. The dietician queries the patient’s diabetes – he might need education. The dietician requests from [resident] “can you refer him to the diabetes clinic?” Resident replies, “Sure. Can he be taught about blood sugar monitoring here?” The dietician says, “The bedside nurse can do that.” A nurse discusses with the dietician the possibility of them both going in together to see the patient for education of blood sugar monitoring (observation, interprofessional round).
2	The resident reports that they are still actively investigating the patient 11. No comments are made. The resident reports that patient 12 is going home today; “it’s all written up and ready to go.” The resident reports that patient 13 aspirated last night and is now in the step up unit. No comments made. The resident reports that patient 14 is also in step up [round continues] (observation, interprofessional round).
3	The charge nurse calls out the patient name but the resident does most of the talking. She asks the allied health staff for information and clarification, they speak up when she asks them something directly (observation, interprofessional round).
4	The physician says to the nurse “should we start?” The nurse replies: “I don’t know where…” The social worker prompts her, and the nurse gets in two sentences before the doctor interrupts and asks the physiotherapist for information. The physician then says “get her out of here,” meaning ‘discharge the patient’ (observation, interprofessional round).
5	The attending physician asks the nurse if there is any news of patient C. “No,” she says. The occupational therapist at the back of the room says quietly, twice, “can we go back to Mr. B?” No one else looks at her or responds, and no one alerts the physician to what she has clearly said too quietly for the attending physician to hear (observation, interprofessional round).
6	“It is intimidating [to speak up at bullet rounds] because everything happens so quickly. The medical team wants a quick discharge and medicine is the focus here because this is acute care. They have pressures on them to discharge. But sometimes, as a result, we don’t get heard in terms of our recommendations for patient care” (interview, occupational therapist).
7	“Bullet rounds have no depth to them. They are too superficial” (interview, nurse).
8	“From the medical perspective, [the problem is that] the information that is shared at bullet rounds is not always useful, like what the functional ability of a patient is” (interview, physician).
9	As they are waiting, the advanced practice nurse enters and comments about the medical staff, “They’re rounding before the round.” Physiotherapist replies, “I guess they want to go over the medical stuff,” but she is clearly a little annoyed with waiting (observation, interprofessional round).

This is an example of how non-physician contributions to an established communication format trigger deliberations that improve patient care in several ways- by identifying a lurking problem hidden by the successfully treated main reason for admission, by defining a speedy discharge process, by improving pre-discharge patient education, and by establishing superior patient follow up. The potentially disrupting medical problem- diabetes- is brought up in a non-threatening fashion by the dietician, which triggers the physician to agree to specialist clinic follow up. Prompted by this mention of the comorbidity, the physician seeks local help with patient education on diabetes to prevent unplanned readmission, and two professions agree to share the task immediately, potentially preventing a delay in discharge. Within less than 15 seconds, an unstructured conversation identifies a problem, agrees on both a multi-facetted solution and on who will implement it, thereby solving a potential discharge problem and likely reducing the risk of readmission.

At both sites the main interprofessional activity was scheduled daily rounds, which were usually chaired by either a physician or a case manager. While these daily gatherings offered the only regular opportunity for interprofessional patient-centred interactions, our data indicated that communication during these rounds were mainly from physicians to nursing and allied health professionals (data extract 2).

Nursing and allied health professionals seldom offered input unless prompted by the chair (data extract 3) or a physician, and interprofessional responses were limited to facts and terse in nature. Indeed, it seemed that no particular planning with regards to effective interprofessional collaboration and communication had gone into the implementation of interprofessional rounds beyond the act of bringing different health professionals together. Consequently, there did not seem to be a clear idea of how effective collaboration would be achieved.

When nursing and allied health professionals participated in the discussion at rounds physicians sometimes ignored their questions (data extract 4) even when those questions were presented as requests for action on the physicians’ part. When nurses or allied health professionals initiated communication, these attempts were often overlooked (data extract 5).

Nursing and allied health indicated that their contributions are ignored during interprofessional rounds (data extract 6), which they feel focus too heavily on immediate medical issues, rather than the functional issues which underpin their work (data extract 7). In a sad mirroring of this mismatch of perspectives, physicians consider other professions’ focus on patient function to be less useful to decisions on care than the physiological information that underpins their own model of care (data extract 8).

Although these rounds were the only scheduled interprofessional communication forum, members of all professions prioritise their own intraprofessional rounds or meetings more highly, with physicians delaying interprofessional rounds and keeping other professionals waiting, while they complete their intraprofessional activities (data extract 9).

The number of unscheduled communications involving physicians was very low. Typically, a physician would not have a single unscheduled communication with a non-physician across many hours of work, a stark contrast to public expectation created by the near-continuous interprofessional conversation, debate and intense exchange of meaningful gazes in medical soap operas on television The excerpts offered are only a small fraction of many such observations (Table 2, data extracts 10, 11, 12).

**Table 2:** Unscheduled interprofessional interactions

10	A nurse calls out, “I need a signature” and she repeats this twice loudly. No one responds (ward observation).
11	A clinical clerk approaches the busy nursing desk from inside the station, flips though some papers and leaves (ward observation
12	The occupational therapist is on the floor as well. I see him as he’s getting onto the elevator and he’s joined by the two young doctors. They don’t speak to one another (ward observation).
13	A medical intern comes in and grabs three charts from the cart. He asks the social worker if any forms need to be filled out for a patient [then leaves] (ward observation).
14	The social worker comes in […] and tells the physiotherapist that she got a bed in a slow stream rehab for a patient. The physiotherapist had talked to the patient’s husband about taking her home. They discuss the decision to be made and agree that it is in the patient’s best interest to go home. The social worker says that she’ll bring it up at rounds (ward observation).
15	“Allied health [are an interprofessional team].We’re more aware of what others do, in terms of having to make referrals. The nurses and doctors don’t have that knowledge. In our professions, the interdisciplinary ones, we’re taught more about what the roles [of other health professionals] are” (interview, social worker).
16	A resident approaches a nurse at the desk and asks her about a patient that she’s seen. The resident then goes to the pharmacist, accompanied by the same nurse, and together they look through what appears to be the patient’s chart (ward observation).
17	At the admin desk the day’s charge nurse is on the phone. A young-looking male doctor comes out of the ICU unit and walks over to her. “Hey Dr. X” she says. “Hey [first name]” he says. They engage in a very friendly chat. “You look good” she says, “you look like you lost weight.” “Really? Thanks,” he says. A second nurse walks over to join them. “So when will you come back to this ward?” the first nurse asks him. He tells her that he’s in neuro now and will only do consults here now. “It’s great to see you guys,” he says (ward observation).
18	“It’s easy to touch base with the other allied professionals on the floor; sometimes it’s crazy to get residents” (interview, social worker).
19	It’s difficult with the nurses.” There are so many and they are often hard to track down. It’s hard to find them in order to speak with them.” [I ask her if she is more often looking to speak to the nurse directly or if she just puts orders in the chart.] “We write a lot of orders, and we change a lot of orders” (interview, resident physician).

When unscheduled interprofessional interactions did occur, they tended to be terse in nature and consist of a question, request or order from the physician to another non-medical colleague (data extract 13). Over roughly 75 hours of unstructured observation time we observed just 14 interactions between physicians on the one hand, and either or both of nursing or allied health professionals.

This is in sharp contrast to unscheduled interactions between nursing and allied health professionals, which were frequent, and more often rich in patient focused content, and characterized by exchange of information and views, and a focus on reaching an agreed-upon course of action (one of many examples is offered in data extract 14). A social worker confirmed at interview that the allied health professions worked together as an effective team, in contrast to the nurses and physicians (data extract 15). We did observe a few unstructured interprofessional interactions involving physicians which contained greater depth (data extract 16), as well as the occasional social exchange (data extract 17), but this type of interprofessional social interaction was extremely rare within this clinical context. Interviews with several health professionals supported our observation that health practitioners engage in limited interaction across professional boundaries (data extracts 18–19):

### Intraprofessional interactions

Outside of the scheduled activities, interprofessional interactions were a rather uncommon feature of work. Even at busy times in the wards, most unscheduled interactions were intraprofessional (Table 3, data extracts 20–21).

**Table 3:** Intraprofessional interactions

20	There’s lots of buzz around the nurses station. A group of nurses and nursing students are standing in a group being coached about filling syringes (ward observation).
21	A man’s leg is being amputated due to gangrene and much discussion takes place between a med student and another young doctor. There is much back and forth about symptoms, cellulitis, potentially life threatening outcomes, and types of meds (ward observation).
22	The palliative care nurse specialist approaches a registered nurse, calling her by her first name, but introducing herself by her specialty only. She’s here to get an update on a patient. They talk for a couple of minutes standing at the counter, and the specialist thanks the registered nurse and leaves (ward observation).
23	Senior resident: “so how’s my amputation?” [Referring to patient whose leg was amputated].
	First-year resident: “okay…but depressed. I ordered a psych consult.”
	Senior resident: “is he wheelchair bound?”
	First-year resident: “No. Well, he’s trying to walk” (shrug).
	[Neither turns to the physiotherapist who is sitting silently beside them] (ward observation).
24	In the hallway two nurses are talking about their sons and how they dress (ward observation).
25	The attending, residents and clerks are chatting casually about the clerks’ going to party last night and being tired today; and whether they’ll be playing the game of medical jeopardy that the chief resident is running tomorrow (ward observation).

Interactions between nurses tended to be rich in content, and were usually characterized by two-way discussion (data extract 22). Physicians’ interactions with other health professionals were usually brief and unidirectional, while their intraprofessional interactions with members of their own profession, although infrequent, involved more dialogue (data extract 23). Beyond offering opportunities to discuss patient-care, unstructured periods of general internal medicine work also provided opportunities for informal social interactions. Such interactions took place primarily on an intraprofessional basis (data extract 24–25):

## Discussion

This qualitative study showed that face-to-face interprofessional communication between physicians and other health professionals in general internal medicine wards were both rare and terse - usually consisting of brief requests for information, or physicians delivering patient-related orders.

Opportunities for interprofessional deliberation on patient care were present but seldom taken up by physicians, and nursing and allied health professionals were often overlooked when they attempted to initiate communication with physicians. This was the case even where the different professions on a team were regularly looking after the same patients due to their ward-based system of organisation (Site A).

Physician disengagement from deliberations on patient care could disadvantage patients in terms of patient and family centred care, and in safety. Our data shows several examples where physician focus on medical/physiological issues could result in a discharge or in-hospital decision that would fail to deal with prospective delays, adverse events or unplanned readmission. This disengagement may also interfere with efficient organisation of patient stays and discharge, and demoralise non-physician staff, contributing to unnecessary frustration and potentially wasteful staff turnover [15].

Physician decision-making seen here excluded potentially useful viewpoints on specific patient care decisions that may originate from other professions. This exclusivity has a long history, in which scientific expertise, and increasing clinical potency separate the status and power of the medical profession as well as the demand for its services; the rising demand (against a background of increasing costs and needs, but relatively static resources) creates pressure to conduct clinical work ever more rapidly, while the status and authority of the profession give it disproportionate weight to select among the questions and prompts it responds to, both at the level of the profession in society, and for each individual physician and their patient care decisions.

Why is the term “teamwork”, with its connotations of open and thoughtful discussion of all viewpoints so prevalent now, when the reality is so different? The widespread use of the term “interdisciplinary team” seems to be rhetorical and represents a hope for the future, rather than a currently attainable reality. Modern interprofessional hospital teams are generally extremely competent and hardworking, polite and respectful to each other and caring with their patients, but physicians are still elevated above the team deliberations. This is troublesome given that evidence indicates that a substantial degree of equality is required for interprofessional collaboration [27,28,39-43]. Sociologist use a framework of status inequality to explain the disparities in remuneration, authority and autonomy [44-46] which we see between physicians and other health professions, and we can use that same framework to explain the much more deliberative communications between nurses and allied health professionals, who are more equal to each other in terms of professional status relative to medicine [47,48].

The comparably rich communication that we observed within medicine -but rarely between medicine and other professions- may mean that physicians do not value expertise outside their discipline partly because they have a limited awareness of others’ knowledge base or scope of professional practice and therefore little personal belief in the value of collaboration, nor a sound grasp of how to collaborate with other professions [49]. Evidence of patient-focused collaboration within professions and between nurses and allied health professionals is encouraging and suggests that knowledge barriers between professions are not immutable.

Although not always touching directly upon patient care, the lack of informal exchanges between doctors and other health professionals may also impact upon the nature of professional relationships on the ward. This is because social interaction is fundamental to the development of trust, meaning and satisfaction in the workplace [50-52]. So long as doctors, nurses, and allied staff continue to interact primarily within the professional solitudes of their own areas of expertise and specialization, there will be limited opportunities for the development of shared values and understandings.

## Conclusion

If we are to deepen the decision-making of interprofessional teams and make use of the skills and observations of most of their members in improving the decisions we make, it is imperative that physicians engage more deeply in deliberations on non-medical issues affecting their patients’ care, notwithstanding the rising pressure of work for physicians, and the educational needs of residents, fellows and medical students. Indeed, in the absence of vast new resources and many more imported physicians, it is likely that high functioning interprofessional teams are the only available route for increasing quality and productivity of our health care system. Interventions aimed at improving communication among team members must take this pressure into account, as well as the real social inequalities that exist between physicians and other health professionals which have for many years limited the scope for high performing teams in health care.

What would such interventions look like? They must cost little (few outside consultants), must not be time consuming (reduces patient care time) and must not be merely rhetorical (few outside consultants, whose use of trite trust and team building exercises is unlikely to make any more difference than it did on previous use). The results must reward all participants with improved workflow and reduced conflict. To date, pay for performance has achieved less than was hoped for, but this is early days, and team based performance-related pay may be worth exploring. Of course, pay for performance requires valid performance measurement of teamwork, which is as yet an unresolved challenge. While there are instruments that allow each team member to rate the quality of their collaboration with other team members, [53] these may be cumbersome on a large scale. Nonetheless, if pay for performance can be thoughtfully designed and transparently and rigorously evaluated, it may be worth exploring this approach.

Other sources of influence, such as educational institutions and health management, will need to promote interventions aimed at engaging physicians in improving communication and collaboration in teams. Interventions on physician communication skill could align with the criteria and processes by which professional licensing bodies evaluate physician collaboration skills and license to practice. These criteria could form part of the hiring, promotion, and credentialing processes in hospitals.

Nurses and allied health professionals’ need new skills to initiate communication with physicians in ways that effectively engage them in deliberation on patient issues that are not medical; for this, detailed research on the discourse of interprofessional teams, identifying successful and failed attempts to engage, will be needed.

This exploratory study formed the basis for the design of an intervention based on equipping all health care team members with communication scripts like those hinted at above. The outcome of the intervention study is explained and discussed in Rice et al. 2010 [54].

### Limitations

There are a number of limitations to this study. Although our findings do resonate with our previous work in similar clinical contexts in both the UK [55] and South Africa [56] further research is needed at other levels of care, with other disciplines and in other countries, to assess the applicability of these findings beyond academic General Internal Medicine wards in urban Canada.

Some might propose that video recording of observation sessions would provide data for a more thorough analysis. While useful in many settings it was not feasible ours due to the busyness of the environment, the intrusiveness of the camera, and the difficulties of de-identification and obtaining informed consent. Issues of reactivity [57,58] -where individuals modify their behaviour because they know they are being observed – may also have influenced our data, as with any observational study. The skill of our researchers in merging into thee setting, combined with the busy nature of ward work have reduced this possibility. To minimize the impact of any of the authors preconceptions, we have used qualitative methods to let the data ‘speak for themselves’, and emerging analyses have been repeatedly discussed among the members of our (interprofessional) research team to reach a shared explanation, rather than allowing any single interpretation to predominate.

Difficulties inherent in assessing collaboration have bearing on this research study. We have used communication as a proxy, and view effective communication as evidence of the intent to collaborate in the interest of good patient care.

## Competing interests

The authors declare that they have no competing interests.

## Authors’ contributions

MZ conceived the project and was involved in all stages, including writing and revising the paper. KR contributed to data-analysis, and to the drafting, and editing of this paper. LGC and KLM contributed to data-collection; LKL, and CK all contributed to the writing and editing this paper. SR contributed to the analysis, and to drafting and editing of this paper. All authors read and approved the final manuscript.

## Pre-publication history

The pre-publication history for this paper can be accessed here:

http://www.biomedcentral.com/1472-6963/13/494/prepub
